# Impact of Residual Compositional Inhomogeneities on the MCT Material Properties for IR Detectors

**DOI:** 10.3390/s24092837

**Published:** 2024-04-29

**Authors:** Jan Sobieski, Małgorzata Kopytko, Kacper Matuszelański, Waldemar Gawron, Józef Piotrowski, Piotr Martyniuk

**Affiliations:** 1 Institute of Applied Physics, Military University of Technology, 2 Kaliskiego St., 00-908 Warsaw, Poland; malgorzata.kopytko@wat.edu.pl (M.K.);; 2 Vigo Photonics, Poznańska 129/133, 05-080 Ożarów Mazowiecki, Polandjpiotr@vigo.com.pl (J.P.)

**Keywords:** HgCdTe, infrared detectors, MOCVD growth, interdiffused multilayer process, residual inhomogeneities, post-growth annealing

## Abstract

HgCdTe is a well-known material for state-of-the-art infrared photodetectors. The interd-iffused multilayer process (IMP) is used for Metal–Organic Chemical Vapor Deposition (MOCVD) of HgCdTe heterostructures, enabling precise control of composition. In this method, alternating HgTe and CdTe layers are deposited, and they homogenize during growth due to interdiffusion, resulting in a near-uniform material. However, the relatively low (350 °C) IMP MOCVD growth temperature may result in significant residual compositional inhomogeneities. In this work, we have investigated the residual inhomogeneities in the IMP-grown HgCdTe layers and their influence on material properties. Significant IMP growth-related oscillations of composition have been revealed in as-grown epilayers with the use of a high-resolution Secondary Ion Mass Spectroscopy (SIMS). The oscillations can be minimized with post-growth annealing of the layers at a temperature exceeding that of growth. The electric and photoelectric characterizations showed a significant reduction in the background doping and an increase in the recombination time, which resulted in dramatic improvement of the spectral responsivity of photoconductors.

## 1. Introduction

Mercury Cadmium Telluride (HgCdTe) is a well-known material for state-of-the-art infrared (IR) photodetectors. It offers continuous wavelength tunability from short-wave infrared (SWIR) to very long-wave infrared (VLWIR), while ensuring high quantum efficiency (QE) and low dark current. However, the fabrication of good-quality IR detectors requires solving technological problems resulting from the instability of the compound and weak ionic bonds. Molecular Beam Epitaxy (MBE) and Metal–Organic Chemical Vapor Deposition (MOCVD) are the two most important techniques for the growth of high-quality HgCdTe heterostructures for IR devices. The interdiffused multilayer process (IMP) for MOCVD growth of Hg_1−x_Cd_x_Te with precise control of cadmium content, *x*, in MOCVD was developed in the 1990s [1]. The process enables to separate the optimization of growth conditions for the CdTe and HgTe layers, which then allow for the growth of high-quality heterostructures. Typically, the MOCVD-grown material shows greater background donor concentration and a shorter recombination time. A possible cause could be not sufficient homogenization of IMP-grown materials during deposition. This applies especially to the upper part of the layer, which is briefly homogenized during growth. The first-to-grow layers are better homogenized than the last ones because of the longer time at the growth temperature. Non-perfect homogenization during IMP leads to the formation of a specific type of 2D superlattice material that consists of thin graded-gap HgCdTe layers with a period equal to the thickness of the IMP CdTe/HgTe pairs (~100 nm). The degree of homogenization is determined by the time of homogenization and the interdiffusion coefficient of Cd and Te in HgCdTe. There have been many articles both experimental and numerical on the interdiffusion coefficient [2,3]. The interdiffusion coefficient depends on composition, x of Hg_1−x_Cd_x_Te, temperature, and Hg pressure. Interdiffusion at temperatures exceeding 450 °C is well described by the model based on fundamental point defect mechanisms [4]. Hg and Cd can diffuse by moving onto interstitial sites or by exchanging places with vacancies on the cation sublattice. The interdiffusion coefficient decreases with decreasing temperature, increasing Cd content and Hg pressure. This is the result of a decreasing concentration of vacancies. The typical IMP growth temperature ranges from 350 °C to 370 °C [5,6,7,8]. The fundamental point defect model does not work well due to the low concentration of vacancies at these temperatures. For this temperature range, there is no reliable experimental data and expressions regarding the interdiffusion coefficient. Interdiffusion and related material composition changes in the near-surface area of the growing layer are in situ monitored using laser reflection interferometry during the IMP process [9]. However, this technique cannot measure the residual compositional gradients in the grown layer. The optical and photoelectrical properties of HgCdTe strongly depend on its composition. Therefore, the residual oscillations of the composition of IMP material may highly modify the properties of photodetectors grown by the IMP technique. The impact of composition oscillations is very complicated (probably negative), determined by many factors and difficult to predict. In this paper, we have measured the changes in compositional profiles of the IMP layers after post-growth annealing at temperatures exceeding that of growth and the impact of the annealing on the photoelectrical properties of the material. Huge IMP growth-related oscillations of iodine dopant concentration were reported [10], evidence of the poor homogenization of the dopant concentration. There are no data in the literature, however, regarding composition fluctuations and their dependence on growth conditions. This is probably caused by too low spatial resolution of the SIMS systems used for microanalysis of the in-depth composition profiles. It was well established that MOCVD-grown Mercury Cadmium Telluride (MCT or HgCdTe) features donor background doping close to 10^15^ cm^−3^ [11], about an order of magnitude higher compared to molecular beam epitaxy (MBE) [12,13]. The preferred orientation of MOCVD-grown HgCdTe film is either the (100) or the (111)B, and each has its advantages and disadvantages. The (100) layers exhibit better crystalline quality and significantly less residual donor concentration in the mid of 10^14^ cm^−3^ [8]. However, the most serious drawback is the presence of surface pyramid-shaped macro-defects known as “hillocks”, which significantly hinder the further processing of detectors. In (111)B epilayers, the background doping is even 10^15^ cm^−3^. The most dominant defects in HgCdTe (111)B epilayers are twin dislocations that exhibit donor-like activity and also result in high surface roughness. Regardless of the higher background doping and roughness, it does not prevent from the obtaining of a background limited infrared photodetector (BLIP) operating in the mid-wave infrared (MWIR) range [14], while there are no hillocks on (111)B layers. Moreover, high p-type doping with arsenic can be achieved in (111)B HgCdTe compared to (100) epilayers. It is especially important for high-operating temperature (HOT) long-wave infrared (LWIR) photodetectors, where high p-type doping is necessary to suppress thermal generation.

Post-growth annealing is a proven method to improve the crystalline quality of MBE-grown HgCdTe. For example, annealing at temperatures below 200 °C of planar devices after ion implantation was studied [15,16]. This sort of annealing is required to activate implanted elements and reduce structural defects after implantation. Post-growth annealing at temperatures from about 200 °C to 300 °C in Hg vapor is commonly used to reduce Hg-vacancy concentration [17]. Reduced dislocation density after post-growth annealing at higher temperatures was reported. Cycle annealing at temperatures between 290 °C and 350 °C, which is close to the MOCVD growth temperature resulted in a reduction in etch pit density (EPD) of HgCdTe grown on CdTe/Si substrate [18]. Surface properties of ion-implanted HgCdTe were studied after annealing at 360 °C [19]. As a result, higher arsenic (acceptor) activation was achieved and extended defects were removed. Degradation of the carrier lifetime was observed after annealing at a temperature of 420 °C in Hg-saturated pressure in H_2_ [20]. The effect was attributed to the creation of an SRH recombination center only when annealing was done in H_2_. On the other hand, in situ annealing between 390 °C and 450 °C in the MBE reactor led to a reduction in EPD [21].

In this paper, we discuss the residual oscillations of cadmium content in IMP-grown (111)B HgCdTe layers and their impact on the background doping and minority carrier lifetime. Furthermore, to remove residual inhomogeneities, the samples were annealed at a temperature of 400 °C for 30 min.

## 2. Materials and Methods

The (111)B HgCdTe samples were grown by the IMP technique in a horizontal Aixtron AIX-200 MOCVD system on 2 inch, epi-ready, semi-insulating (100) GaAs substrates (AXT Inc., Beijing, China) buffered with a 3 μm thick CdTe layer to compensate the lattice mismatch between the GaAs substrate and the HgCdTe layer. The growth was carried out at a temperature of approximately 350 °C with the mercury zone at a temperature of 170 °C. Diisopropyltelluride (DIPTe) and dimethylcadmium (DMCd) (Dockweiler Chemicals GmbH, Marburg, Germany) were used as precursors for Te and Cd, respectively. Elemental mercury (Alfa Aesar Thermofisher Haverhill, Massachusetts, United States) was used as a mercury precursor, and H_2_ was used as the carrier gas. Two non-intentionally doped (n.i.d.) HgCdTe samples were prepared for the research as follows:

- Sample MW with a planned x = 0.29 at a cutoff wavelength of 4.5 μm at 300 K,
- Sample SW with a planned x = 0.37 at a cutoff wavelength of 3.2 μm at 300 K.

After the growth, the samples were annealed for 30 min at a temperature of 400 °C in mercury pressure of 50 mbar and then slowly cooled down to 150 °C in varying near-saturated Hg vapor pressure. Secondary ion mass spectrometry (SIMS) experiments were performed with the CAMECA IMS SC Ultra instrument to identify composition inhomogeneities. Cesium was used as a primary ion source and with the positive detector polarity all signals were registered as CsX^+^ cluster ions. The impact energy, intensity, and beam diameter were 100 eV, 0.5 nA, and 20 microns, respectively. The impact angle was increased to 79°. Such non-trivial conditions practically eliminated the mixing effect and, in some cases, ensured the atomic depth resolution [22]. It allowed us to detect even small fluctuations in the chemical composition which would not be possible in the classical SIMS experiment (i.e., with much higher impact energy and the impact angle in the range of 40–60°). X-ray diffraction rocking curves were collected with PANalytical X’Pert—HRXRD. Full Width at Half Maximum of the peak from (511) plane was used to reveal the changes of layers crystalline quality due to the 400 °C anneal. Electrical properties were estimated by the Hall effect measurements at a temperature range from 80 K to 300 K using the Van der Pauw technique with a 0.542 T magnetic field. The minority carrier lifetime was measured by photoconductivity decay method using a 1 mm long photoconductive device illuminated by a 1.6 μm wavelength pulse laser. The same devices were used for the spectral current responsivity measurement using the PerkinElmer Spectrum 3 FTIR spectrophotometer.

## 3. Results and Discussion

**Figure 1:**
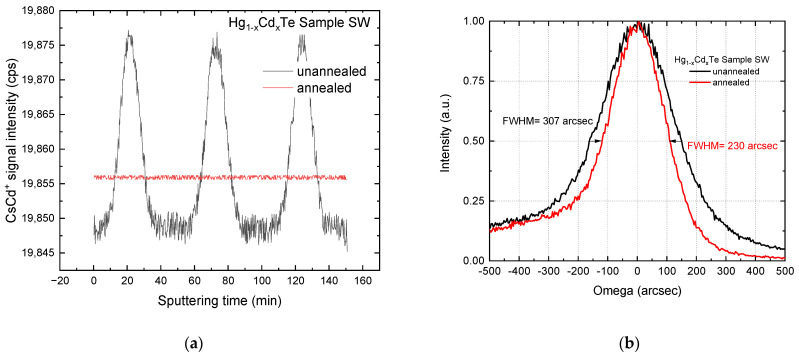
The relative Cd SIMS intensity (**a**) of and XRD rocking curves (**b**) of the as-grown and annealed Hg_1−x_Cd_x_Te (x = 0.37) layers.

Figure 1 shows the SIMS composition measurements of the as-grown sample and the sample subjected to the post-growth annealing at temperatures higher than the growth temperature. Significant oscillations of composition can be seen. The period of oscillations equal to the thickness of the IMP HgTe/CdTe pairs was found. In contrast, no oscillations of Cd-related SIMS signal intensity at any part of the epilayer can be seen in the sample subjected to the post-growth annealing. This shows a practically perfect IMP layer homogenization during the 30 min anneal at 400 °C. This is also confirmed by a significant narrowing of the FWHM of the x-ray diffraction rocking curve from 307 arcsec to 230 arcsec. The relatively large FWHM is probably due to the design of the sample structure that also consists of the CdTe buffer and its interface of graded composition with the HgCdTe epilayer.

The IMP layers grown at relatively low temperatures without post-growth annealing can be treated as a long (~100 nm) period superlattice of variable bandgap HgCdTe pairs. This may affect the electric and photoelectric properties of the material.

Relations on energy gap [23], carrier mobilities [24], minority carrier lifetime [25], composition for Hg_1−x_Cd_x_Te are well established. The composition of samples was determined by measurements of the spectral photoconductivity of the samples [26]. Minority carrier lifetime was measured by the photoconductivity time response method [27].

**Figure 2:**
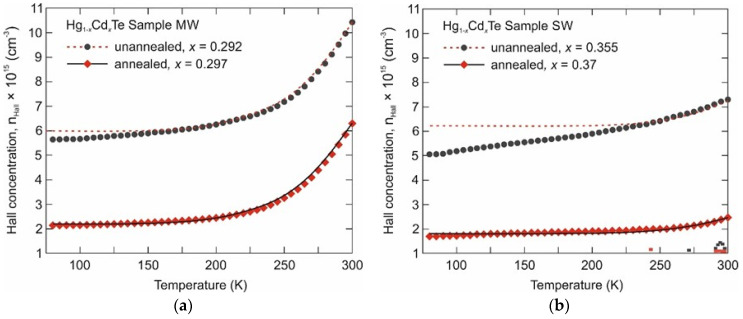
Hall electron concentration versus temperature for the Hg_1−x_Cd_x_Te Sample MW (**a**) and Sample SW (**b**), measured after growth and after additional annealing. Experimental results (points) and fitted calculated lines for homogeneous layer.

Figure 2 shows the measured and calculated Hall carrier concentrations. Measured at 80 K, the Hall concentrations for as-grown and annealed MW and SW samples were, respectively, as follows:

- MW: ≈ 5.5 × 10^15^ cm^−3^ and 2.2 × 10^15^ cm^−3^,
- SW: ≈ 5 × 10^15^ cm^−3^ and 1.9 × 10^15^ cm^−3^.

For calculated Hall concentrations, uniform compositions (Table 1) were assumed. Donor concentrations for as-grown and annealed MW and SW samples were taken, respectively, as follows:

- MW: ≈ 6.0 × 10^15^ cm^−3^ and 2.2 × 10^15^ cm^−3^,
- SW: ≈ 6.2 × 10^15^ cm^−3^ and 1.9 × 10^15^ cm^−3^.

The results show a significant reduction in the background donor concentrations after the homogenization annealing. It should be noted that the measured and calculated Hall concentrations are in good agreement for the annealed samples. In contrast, there is a large discrepancy in the low-temperature range between the experimental and calculated concentrations. This is due to the oscillations of material composition not being taken into account in the calculations. The nature of the high Hall concentrations in as-grown samples is not clear yet. This may be caused by the composition gradients themselves, specific properties of the superlattice material structure or defects of the material. The annealing may eliminate all the reasons.

**Figure 3:**
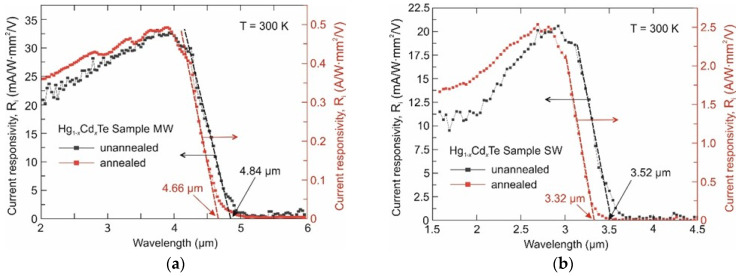
Spectral current responsivity of the photoconductive detectors fabricated from the as-grown and 400 °C annealed Sample MW (**a**) and Sample SW (**b**).

Figure 3 shows the normalized spectral responsivities of photoconductors fabricated from the as-grown and annealed HgCdTe layers. The annealing results in two important changes as follows:

- Blue shift of the long wavelength photoresponse cutoff. This is probably due to the material homogenization that leads to an increase in the bandgap in HgTe-rich parts of the post-IMP periodic structures,
- Dramatic increase in the responsivity by much more than one order of magnitude.

This is the result of the increase in the materials’ electron mobility and recombination time product (μ_e_ × τ). The homogenization probably increases both μ_e_ and τ. This would improve the performance of not only photoconductors but also other types of IR photodetectors.

**Figure 4:**
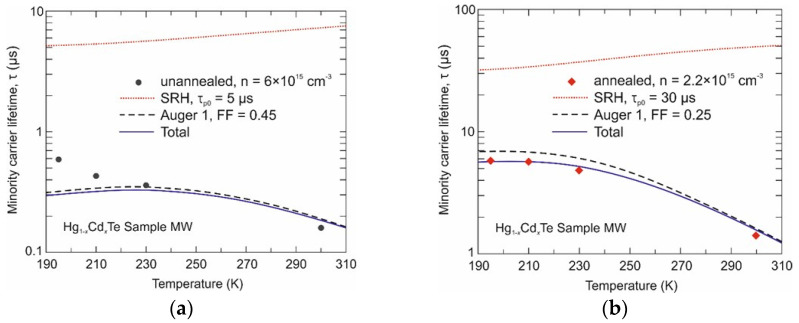
Measured and calculated minority carrier lifetime versus temperature for the Hg_1−x_Cd_x_Te Sample MW layer after growth (**a**) and after additional annealing (**b**).

**Figure 5:**
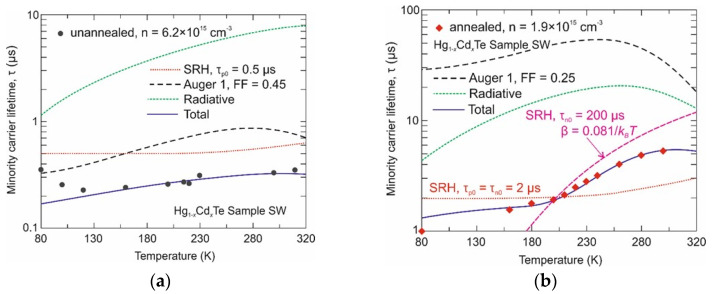
Measured and calculated minority carrier lifetime versus temperature for the Hg_1−x_Cd_x_Te Sample SW after growth (**a**) and after additional annealing (**b**).

Figure 4 and Figure 5 show the experimental dependence of minority carrier lifetimes on temperature for the Hg_1−x_Cd_x_Te epilayer with *x* = 0.29 (Sample MW) and *x* = 0.37 (Sample SW), respectively. The evolution of time response with temperature is characteristic of each recombination mechanism. Radiative, Auger 1, and non-fundamental Shockley–Read–Hall (SRH) recombination processes were considered in the theoretical analysis (see Appendix A). For the unannealed Sample MW (with residual composition inhomogeneities), the minority carrier lifetime is limited by the Auger 1 recombination. This is probably due to high intrinsic concentration at high temperatures and high background donor doping at temperatures < 250 K. In the case of the unannealed Sample SW, all three effects determined the minority carrier lifetime. Discrepancies between the theoretical results and those calculated at lower temperatures may result from similar discrepancies in electron concentration (see Figure 2). It has to be noted that theoretical analysis assumes uniform material and thus may not be sufficient for the material with residual compositional inhomogeneities. Auger 1 recombination strongly depends on electron concentration and thus it is expected to be partially suppressed by lower background doping after additional samples’ annealing. In the narrow bandgap material (Sample MW), the intrinsic concentration at high temperature is significantly higher than the background doping. Thus, the increase in carrier lifetime due to composition homogenization is not only due to reduced background donor doping (increased Auger 1 lifetime) but mainly due to a significant increase in the SRH lifetime. After additional annealing, τ_p0_ increases from 5 μs to 30 μs. The SRH mechanism determines the carrier lifetime in the annealed Sample SW and is much shorter (by an order of magnitude) than in the annealed Sample MW. However, what is worth pointing out is that it has a strong temperature dependence. Thus far, the SRH lifetime calculated using Equation (A8) is valid only for neutral defects, i.e., when τ_p0_ = τ_n0_. This approach causes an underestimation of the minority carrier lifetime temperature > 200 K (Figure 5b). A good agreement of the total carrier lifetime was obtained, assuming a temperature-dependent SRH lifetime above 200 K. According to Equation (A10), τ_n0_ >> τ_p0_ and equals to 200 μs, and the effective energy trap level lies of about 81 meV above the edge of the valence band. In an *n*-type semiconductor, the SRH lifetime is temperature dependent for strongly charged acceptor defects [28].

## 4. Conclusions

In summary, the residual in-depth oscillations of composition in the HgCdTe layers grown by the IMP MOCVD epitaxy and their influence on the photoelectric properties of the material were studied. Significant in-depth oscillations of composition are easily observable in the SWIR and MWIR epilayers grown at 350 °C with high-resolution in-depth SIMS measurements. The oscillations disappear with the 400 °C annealing for 30 min, indicating practically complete homogenization of the material. The annealing highly improves the structural and photoelectric properties of the layers and can be used to manufacture infrared photodetectors. Incomplete homogenization of IMP pairs results in increased background doping, decreased carrier lifetime, and a red shift in the absorption edge.

## Figures and Tables

**Table 1:** Summary of measured parameters. Values in brackets correspond to samples after annealing.

Parameter	SW	MW
x	0.355	0.292
	(0.370)	(0.297)
λ_cut-off_ (300 K)	3.52 μm	4.84 μm
	(3.32 μm)	(4.66 μm)
XRD rocking curve FWHM	307 arcsec	-
	(230 arcsec)	-
SRH lifetime	0.5 μs	5 μs
	(2 μs) τ_p0_ = τ_n0_ (200 μs) τ_n0_ >> τ_p0_	(30 μs)
Total lifetime at 300 K	0.73 μs	0.15 μs
	(4.84 μs)	(1.41 μs)
Hall concentration at 80 K	5.0 × 10^15^ cm^−3^	5.5 × 10^15^ cm^−3^
	(1.9 × 10^15^ cm^−3^)	(2.2 × 10^15^ cm^−3^)

## Data Availability

The original contributions presented in the study are included in the article material, further inquiries can be directed to the corresponding author/s.

## Appendix A

The radiative carrier lifetime is given by van Roosbroeck and Schockley expression [29]:

(A1) $$\tau_{R}=\frac{1}{B\left(n_{0}+p_{0}\right)},$$

where *n*_0_ and *p*_0_ are the equilibrium electron and hole concentrations, respectively, and

(A2) $$B=5.8\cdot 10^{-13}{\varepsilon_{\infty }}^{\frac{1}{2}}{\left(\frac{m_{0}}{m_{e}^{*}+m_{hh}^{*}}\right)}^{\frac{3}{2}}\left(1+\frac{m_{0}}{m_{e}^{*}}+\frac{m_{0}}{m_{hh}^{*}}\right){\left(\frac{300}{T}\right)}^{\frac{3}{2}}E_{g}^{2}.$$

$\varepsilon_{\infty }$ is the high frequency permittivity, *m*_0_ is the free electron mass, where $m_{e}^{*}$ and $m_{hh}^{*}$ are the electron and hole effective mass, respectively.

*E_g_* is the semiconductor energy gap at a temperature *T*. For Hg_1−x_Cd*_x_*Te, the energy gap is given by the empirical relation [30]:

(A3) $$E_{g}\left(x,T\right)=-0.302+1.93x-0.81x^{2}+0.832x^{3}+5.35\cdot 10^{-4}(1-2x)T,$$

where *x* is Cd molar composition.

For the *n*-type material, Auger 1 is the primary mechanism due to the interaction of two electrons and one hole, and is given by

(A4) $$\tau_{A1}=\frac{2\tau_{Ai1}n_{i}^{2}}{n_{0}\left(n_{0}+p_{0}\right)},$$

where *n_i_* is the intrinsic carrier concentration, for Hg_1−x_Cd*_x_*Te given by [30]:

(A5) $$\begin{array}{c} n_{i}=\left(5.585-3.82x+1.753\cdot 10^{-3}T+1.364\cdot 10^{-3}xT\right)\times \\ \times 10^{14}E_{g}^{3/4}T^{3/2}\exp\left(-\frac{E_{g}}{2k_{B}T}\right) \end{array},$$

*τ_Ai1_* is the Auger 1 lifetime in intrinsic material [31]:

(A6) $$\tau_{Ai1}=3.8\cdot 10^{-18}\varepsilon^{2}{\left(1+\mu \right)}^{\frac{1}{2}}\left(1+2\mu \right)\left(\frac{m_{0}}{m_{e}^{*}}\right)exp\left(\frac{-q\left(1+2\mu \right)E_{g}}{\left(1+\mu \right)k_{B}T}\right){\left|FF\right|}^{-2}{\left(\frac{k_{B}T}{qE_{g}}\right)}^{-\frac{3}{2}},$$

where *k_B_* is the Boltzmann constant, ε is the dielectric constant, *q* stands for the electron charge, *µ* must be taken as $m_{e}^{*}/m_{hh}^{*}$, and *FF* is the overlap integral for Bloch functions.

The carrier lifetime associated with a single SRH defect level is expressed as [32,33]:

(A7) $$\tau_{SRH}=\frac{\tau_{p0}\left(n_{0}+n_{1}\right)+\tau_{n0}\left(p_{0}+p_{1}\right)}{n_{0}+p_{0}}.$$

where *τ_p_*_0_ and *τ_n_*_0_ are the defect capture time constants for electrons and holes.

(A8a) $$n_{1}=N_{c}exp\left[\frac{\left(E_{T}-E_{c}\right)}{k_{B}T}\right],$$

(A8b) $$p_{1}=N_{v}exp\left[\frac{-\left(E_{T}-E_{v}\right)}{k_{B}T}\right],$$

where *N_c_* and *N_v_* are the densities of states in the conduction band and valence band, respectively. *E_T_* is the effective energy level of traps, and *E_c_* and *E_v_* are the energies of the conduction band and valence band, respectively.

The optimum thermal transition, i.e., the shortest SRH lifetime, occurs through defects located approximately at the intrinsic energy level in the semiconductor bandgap, where *n*_1_ = *p*_1_ = *n_i_*. Assuming the same defect capture time constants for electrons and holes, *τ_p_*_0_ = *τ_n_*_0_, Equation (A7) in *n*-type semiconductor becomes:

(A9) $$\tau_{SRH}=\frac{\tau_{p0}\left(n_{0}+2n_{i}\right)}{n_{0}}.$$

At low temperatures, where *n*_0_ > *n_i_*, *τ_SRH_* = *τ_p_*_0_. At high temperatures, where *n*_0_ = *p*_0_ = *n_i_*, *τ_SRH_* = 2*τ_p_*_0_. So can be seen, for the neutral SRH centers, *τ_SRH_* is nearly temperature independent.

The SRH lifetime can be large when *E_T_* does not coincide with *E_i_*. For example, for charged acceptor-type defects in *n*-type semiconductor, where *τ_p_*_0_ << *τ_n_*_0_, the SRH lifetime becomes:

(A10) $$\tau_{SRH}\sim \tau_{n0}exp\left[\frac{-\left(E_{T}-E_{v}\right)}{k_{B}T}\right]{=\tau }_{n0}exp\left[-\beta \right].$$

Considering all three mechanisms, the total minority carrier lifetime in *n*-type semiconductor is given as

(A11) $$\frac{1}{\tau }=\frac{1}{\tau_{R}}+\frac{1}{\tau_{A1}}+\frac{1}{\tau_{SRH}}$$

## References

[1] Irvine S., Mullin J. (1981). The growth by MOVPE and characterisation of Cd_x_Hg_1−x_Te. J. Cryst. Growth.

[2] Zanio K., Massopust T. (1986). Interdiffusion in HgCdTe/CdTe structures. J. Electron. Mater..

[3] Tang M.S., Stevenson D.A. (1987). Interdiffusion behaviour of HgTe-CdTe junctions. Appl. Phys. Lett..

[4] Holander-Gleixner S., Robinson H., Helms C. (1998). Simulation of HgTe/CdTe interdiffusion using fundamental point defect mechanisms. J. Electron. Mater..

[5] Piotrowski A., Madejczyk P., Gawron W., Klos K., Pawluczyk J., Rutkowski J., Piotrowski J., Rogalski A. (2007). Progress in MOCVD growth of HgCdTe heterostructures for uncooled infrared photodetectors. Infrared Phys. Technol..

[6] Bevan M., Greggi J., Doyle N. (1990). Structural studies of HgCdTe grown by MOCVD on lattice-matched substrates. J. Mater. Res..

[7] Hoke W.E., Traczewski R. (1983). Metal-organic vapor deposition of CdTe and HgCdTe films. J. Appl. Phys..

[8] Schmit J.L. (1985). MOCVD growth of CdTe and HgCdTe. J. Vac. Sci. Technol. A.

[9] Irvine S., Bajaj J., Sankur H. (1992). Complete in situ laser monitoring of MOCVD HgCdTe/CdTe/ZnTe growth onto GaAs substrates. J. Cryst. Growth.

[10] Maxey C., Jones C., Metcalfe N. (1996). Growth of fully doped HgCdTe heterostructures using a novel iodine doping source to achieve improved device performance at elevated temperatures. J. Electron. Mater..

[11] Izhnin I., Mynbaev K., Voitsekhovsky A., Korotaev A., Fitsych O., Pociask-Bialy M., Dvoretsky S. (2015). Background donor concentration in HgCdTe. Opto-Electron. Rev..

[12] Wu O.K., Kamath G.S. (1991). An overview of HgCdTe MBE technology. Semicond. Sci. Technol..

[13] Rogalski A., Kopytko M., Martyniuk P., Hu W. (2020). Comparison of performance limits of HOT HgCdTe photodiodes with 2D material infrared photodetectors. Opto-Electron. Rev..

[14] Kopytko M., Keblowski A., Gawron W., Pusz W. (2016). LWIR HgCdTe barrier photodiode with Auger-suppression. Semicond. Sci. Technol..

[15] Yang L., Guo H., Shen C., Xie H., Yang D., Zhu L., Wang F., Sun Q., Chen L., Lin C. (2022). Modeling and characteristics of MWIR HgCdTe APD at different post-annealing processes. Infrared Phys. Technol..

[16] Ajisawa A., Oda N. (1995). Improvement in HgCdTe Diode Characteristics by Low Temperature Post-Implantation Annealing. J. Electron. Mater..

[17] Attolin G., De Melo O., Leccabue F., Panizzieri R., Pelosi C. (1989). Isovpe growth, post-growth annealing and characterization of Hg1−xCdxTe layers. Mater. Lett..

[18] Simingalam S., Brill G., Wijewarnasuriya P., Rao M.V. (2015). Low Temperature, Rapid Thermal Cycle Annealing of HgCdTe Grown on CdTe/Si. J. Electron. Mater..

[19] Korotaev A.G., Izhnin I.I., Mynbaev K.D., Voitsekhovskii A.V., Nesmelov S.N., Dzyadukh S.M., Fitsych O.I., Varavin V.S., Dvoretsky S.A., Mikhailov N.N. (2020). Hall-effect studies of modification of HgCdTe surface properties with ion implantation and thermal annealing. Surf. Coat. Technol..

[20] Edwall D.D., DeWames R.E., McLevige W.V., Pasko J.G., Arias J.M. (1998). Measurement of minority carrier lifetime in n-type MBE HgCdTe and its dependence on annealing. J. Electron. Mater..

[21] He L., Wang S.L., Yang J.R., Yu M.F., Wu Y., Chen X.Q., Fang W.Z., Qiao Y.M., Gui Y., Chu J. (1999). Molecular beam epitaxy (MBE) in situ high-temperature annealing of HgCdTe. J. Cryst. Growth.

[22] Michalowski P., Anayee M., Mathis T.S., Kozdra S., Wójcik A., Hantanasirisakul K., Jóźwik I., Piątkowska A., Możdżonek M., Malinowska A. (2022). Oxycarbide MXenes and MAX phases identification using monoatomic layer-by-layer analysis with ultralow-energy secondary-ion mass spectrometry. Nat. Nanotechnol..

[23] Hansen G.L., Schmit J., Casselman T. (1982). Energy gap versus alloy composition and temperature in Hg_1−x_Cd_x_Te. J. Appl. Phys..

[24] Najafi Bavani S., Akhoundi Khezrabad M. (2021). The electron mobility in HgCdTe (x = 0.22 and 0.3): A comparison between experimental and theoretical results. Mater. Res. Bull..

[25] Jozwikowski K., Kopytko M., Rogalski A. (2011). Numerical estimations of carrier generation-recombination processes and photon recycling effect in 3 μm n-on-p HgCdTe photodiodes. Opt. Eng..

[26] Anderson W. (1980). Absorption constant of PbSnTe and HgCdTe alloys. Infrared Phys..

[27] Kopytko M., Sobieski J., Gawron W., Keblowski A., Piotrowski J. (2021). Minority carrier lifetime in HgCdTe(100) epilayers and their potential application to background radiation limited MWIR photodiodes. Semicond. Sci. Technol..

[28] Kinch M.A. (2014). State-of-the-Art Infrared Detector Technology.

[29] Van Roosbroeck W., Shockley W. (1954). Photon-Radiative Recombination of Electrons and Holes in Germanium. Phys. Rev..

[30] Hansen G.L., Schmit J.L. (1983). Calculation of intrinsic carrier concentration in HgCdTe. J. Appl. Phys..

[31] Beattie A.R., Landsberg P.T., Fröhlich H. (1959). Auger effect in semiconductors. Proc. R. Soc. Lond. Ser. A Math. Phys. Sci..

[32] Shockley W., Read W.T. (1952). Statistics of the Recombinations of Holes and Electrons. Phys. Rev..

[33] Hall R.N. (1952). Electron-Hole Recombination in Germanium. Phys. Rev..

